# Capacitive Biosensors and Molecularly Imprinted Electrodes

**DOI:** 10.3390/s17020390

**Published:** 2017-02-17

**Authors:** Gizem Ertürk, Bo Mattiasson

**Affiliations:** 1 CapSenze Biosystems AB, Lund 223 63, Sweden; bo.mattiasson@biotek.lu.se; 2 Department of Biotechnology, Lund University, Lund 222 40, Sweden

**Keywords:** capacitive biosensors, affinity biosensors, microcontact imprinting

## Abstract

Capacitive biosensors belong to the group of affinity biosensors that operate by registering direct binding between the sensor surface and the target molecule. This type of biosensors measures the changes in dielectric properties and/or thickness of the dielectric layer at the electrolyte/electrode interface. Capacitive biosensors have so far been successfully used for detection of proteins, nucleotides, heavy metals, saccharides, small organic molecules and microbial cells. In recent years, the microcontact imprinting method has been used to create very sensitive and selective biorecognition cavities on surfaces of capacitive electrodes. This chapter summarizes the principle and different applications of capacitive biosensors with an emphasis on microcontact imprinting method with its recent capacitive biosensor applications.

## 1. Introduction

Affinity biosensors can be divided into two main groups: those that measure direct binding between the target molecule and the affinity surface on the sensor, and those biosensors which are adopted to binding assays using labelled reagents [1]. Biosensors operating with labelled affinity-reagents are variations of conventional immunoassay technology in which fluorescent markers, active enzymes, magnetic beads, radioactive species or quantum dots are generally used as labelling agents to label the target molecules [2,3].

Labelling is generally used to significantly facilitate the signal generation and to confirm the interaction between the probe and target molecules [4]. An important feature in using labelled reagents is the amplification of the registered signals and thereby also the sensitivity one can reach. Assays based on the use of labelled reagents are time consuming and the labelled reagents are often expensive. Furthermore, assays with labelled reagents are usually multistep processes and that limit their application for real-time measurements.

The design of label-free affinity based biosensors is the objective of much current research. The aims are to establish alternative methods to the commercial ELISA-based immunoassays. The most attractive features of these types of biosensors are that they allow the monitoring of the analytes directly and in real-time [5].

Biosensors can be divided into four main groups according to transducer types [6]. These main groups involve: electrochemical transducers which involve potentiometric, voltammetric, conductometric, impedimetric and field-effect transistors; optical transducers which include surface plasmon resonance (SPR) biosensors; piezoelectric transducers to which quartz crystal microbalance (QCM) biosensors can be given as an example; and thermometric transducers which measure the amount of heat with a sensitive thermistor to determine the analyte concentration.

Among the different types of label-free biosensors, electrochemical biosensors have received particular attention owing to their properties [4]. These biosensors can also be miniaturized which is very important for many applications that need portable integrated systems. Miniaturization not only allows use at point of care, in a clinic, doctor’s office or at home but also reduces the cost of the diagnostic assays. Electrical biosensors fulfil these purposes as being fast, cheap, portable, miniaturized and label-free devices. Electrical biosensors can be classified as amperometric, voltametric impedance or capacitive sensors.

This review deals with capacitive biosensors, different applications of capacitive biosensors developed for both detection of various targets and by using molecular imprinting technology with an emphasis on microcontact imprinting method. In this aspect, this is a novel review which includes the two technologies, capacitive biosensors and molecular imprinting; at the same time with lots of examples from published reports. The reports in molecular imprinting section were selected mainly from capacitive biosensors developed by using microcontact imprinting method.

## 2. Capacitive Biosensors

Capacitive biosensors belong to the sub-category of impedance biosensors [3]. Capacitive biosensors measure the change in dielectric properties and/or thickness of the dielectric layer at the electrolyte-electrode interface when an analyte interacts with the receptor which is immobilized on the insulating dielectric layer [1].

The electric capacitance between the working electrode (an electrolytic capacitor/the first plate) and the electrolyte (the second plate) is given by Equation (1) [7]:

C = (ε_0_εA)/d (1)

where ε is the dielectric constant of the medium between plates, ε_0_ is the permittivity of the free space (8.85 × 10^−12^ F/m), A is the surface area of the plates (m^2^) and d is the thickness of the insulating layer (m).

According to the given equation above, when the distance between the plates increases, the total capacitance decreases. In other words, in the assaying principle of this type of capacitive biosensors when a target molecule binds to the receptor, displacement of the counter ions around the capacitive electrode results in a decrease in the capacitance. The higher the amount of target molecules bound to the receptor is, the greater is the achieved displacement and the decrease in the registered capacitance [8].

**Figure 1:**
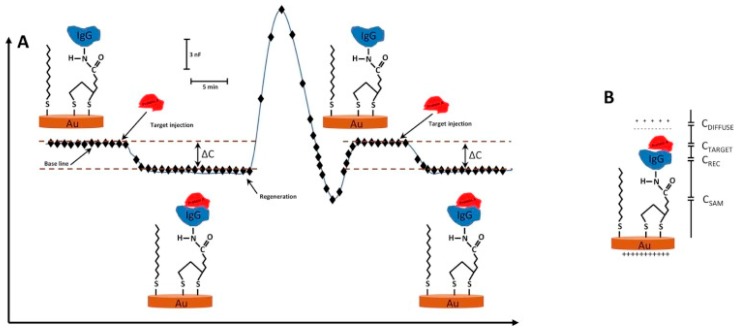
(**A**) Schematic diagram showing the change in capacitance (ΔC) as a function of time when the analyte (IgG) interacts with the receptor molecule (Protein A) immobilized on the surface of the electrode. Subsequent rise in signal is due to the dissociation after the injection of the regeneration solution. In an ideal sensorgram, the baseline should turn back to the original level after regeneration of the surface; (**B**) Immobilization of the receptor molecule on the transducer surface via a self-assembled monolayer (SAM) of alkylthiols. When the target molecule interacts with the receptor, this creates a double layer of counter ions around the gold transducer which results in a change in the capacitance. (Reproduced from Reference [8] with permission).

The assaying principle of capacitive biosensors which are developed according to this rule is shown in Figure 1.

The Equation (1) can be represented by two capacitors in series where the inner part includes the dielectric layer (C_dl_) and the outer one corresponds to the biomolecule layer (C_bm_). Then, the total capacitance (C_t_) can be described as Equation (2) [7].

(2) $$\frac{1}{C_{t}}=\frac{1}{C_{\mathrm{dl}}}+\frac{1}{C_{\mathrm{bm}}}$$

The electrochemical capacitors which are described based on the above-mentioned equation are known as constant phase element (CPE). The presence of CPE indicates that the observed capacitance of the system is frequency dependent.

Capacitance can also be defined as Equation (3) [7].

(3) $$Z=\frac{1}{\omega C}$$

where Z is the impedance and ω is the radial frequency expressed in rad·s^−1^. This model implies that all of the measured current is capacitive.

## 3. Different Applications of Capacitive Biosensors

Different applications of capacitive biosensors developed for different targets are summarized in Table 1.

### 3.1. Protein Detection

Labib et al. [9] developed a sensitive method for detection of cholera toxin (CT) using a flow-injection capacitive immunosensor based on self-assembled monolayers. Monoclonal antibodies against the subunit of CT (anti-CT) were immobilized on the gold electrode surface which was modified by lipoic acid and 1-Ethyl-3-(3-dimethylaminopropyl)carbodiimide (EDC). The immunosensor showed linear response to CT concentrations in the concentration range between 1.0 × 10^−13^ M and 1.0 × 10^−10^ M under optimized conditions. Limit of detection (LOD) value was 1.0 × 10^−14^ M. The LOD value obtained from capacitive immunosensor was compared with the LOD values that were obtained from sandwich ELISA and SPR based immunosensors. The ELISA had a LOD of 1.2 × 10^−12^ M whereas SPR had a LOD value of 1.0 × 10^−11^ M. The results proved that the method is more sensitive than the other two techniques used in this study.

In another study by the same research group, a label-free capacitive immunosensor was developed for direct detection of CT present at sub-attomolar level. Gold nanoparticles (AuNPs) were incorporated on a polytyramine modified gold electrode and anti-CT antibody was immobilized on this surface. Tyramine provides free amino groups which are very useful to immobilize the affinity ligand to the transducer. At the same time very thin and uniform films can be formed on the electrode surfaces during the electro-polymerization of tyramine. After the immobilization step, the formation of antigen-antibody complexes resulted in a change in capacitance and by this way the concentration of CT was determined. The dynamic range was between 0.1 aM and 10 pM where the LOD value was 9 × 10^−20^ M (0.09 aM). The electrode could be regenerated with a good reproducibility for up to 36 times with a relative standard deviation (RSD) value of 2.5%. Real sample analyses were performed from water samples collected from a local stream and matrix effect was eliminated with a 10.000 times dilution prior to analysis. The developed system had potential to be used as a portable electrochemical analyser for field conditions [10].

The same strategy was used to develop a capacitive biosensor for sensitive detection of HIV-1 p24 antigen [11]. Following polytyramine electro-polymerization on the gold electrode surface, gold nanoparticles were incorporated onto the electrode and then, anti-HIV-1 p24 monoclonal antibodies were immobilized on top. HIV-1 p24 antigen was detected from standard p24 solutions in the concentration range of 10.1 × 10^−20^ to 10.1 × 10^−17^ M. The reasons for extreme sensitivity of the capacitive biosensors were explained by the authors in two ways. Firstly, the capacitive technique is very convenient to detect the size and position of the electrical double layer formed at the interface. However, in other electrochemical techniques such as the amperometric technique, the detection is based on the measurement of only the transport of electrons. The second factor is the increased surface area on the electrode via the use of immobilized AuNPs. The gold nanoparticles also significantly contributed to the decrease in the interfacial resistance which facilitated the electron transfer at the electrode surface [11].

Qureshi et al. [12] developed a capacitive aptamer based sensor for detection of vascular endothelial growth factor (VEGF) in human serum. Systematic evolution of ligands by exponential enrichment (SELEX) process was utilized to select the highly specific and selective anti-VEGF aptamer to bind VEGF to the aptasensor surface. When a sandwich assay was tested by forming sandwich complex with anti-VEGF aptamer+VEGF+anti-VEGF antibody, the generated signal was enhanced by 3–8 folds compared to the direct assay. The developed sensor showed a dynamic detection range from 13 × 10^−14^ M to 2.6 × 10^−11^ M of VEGF protein in human serum. The results showed that the developed system could be successfully used in clinical diagnosis to detect biomarkers in real samples in a convenient and sensitive way.

### 3.2. Nucleic Acid Detection

Mahadhy et al. [26] used in a model study the capacitive sensor to monitor the capture of complementary single-stranded nucleic acids. A 25-mer oligo-C was immobilized onto the polytyramine modified gold electrode surface. Temperature was raised to 50 °C to reduce non-specific hybridization in order to increase the selectivity and hybridization was used in order to amplify the signal by using longer nucleic acid molecules.

Later, Mahadhy et al. [13] developed a promising ultrasensitive, automated flow-based and portable gene sensor. The PCR-free biosensor proved the possibility for powerful detection of foodborne pathogens in diagnostic situations and multi-drug resistant bacteria in the near future. Rapid detection of foodborne pathogens is crucial before many people are infected while early detection of multi-drug resistant bacteria is important to isolate the infected patients earlier and to reduce the risk of spreading.

Two functionalization layers; gold (Au) and 3-glycidoxypropyl-tri-methoxy silane (GOPTS) were used to immobilize thiol modified oligonucleotides on silicon surfaces. GOPTS showed better performance as a functionalization layer because the hybridization efficiency was higher, the stability over time was better and regeneration of the surface after analyte binding was easier. Therefore for the development of microcantilever or micro-membrane based biosensors, GOPTS might be a more promising alternative [14].

A single stranded DNA (ssDNA) aptamer was developed to bind to nicotinamide phosphoribosyl transferase (Nampt) through SELEX and implemented in a capacitive biosensor [15]. The LOD for Nampt was 1.8 × 10^−11^ M with a dynamic detection range in serum of up to 9 × 10^−10^ M. Nampt is an important biomarker for obesity-related metabolic diseases, some types of cancers and chronic diseases. Normal level of Nampt in human plasma is around 15 ng·mL^−1^ (27 × 10^−11^ M). Therefore, the developed system has potential as a diagnostic tool and in point-of-care applications.

Pyrrolidinyl peptide nucleic acid probes were immobilized onto the self-assembled monolayer (SAM) modified gold electrode surface to develop a DNA capacitive biosensor [16]. Four different alkanethiols with various chain lengths were used as a SAM to determine the influence of the length and the terminating head group of blocking thiols on the sensitivity and specificity. In the study, the blocking thiol which had an equal length to the –OH terminating head group gave the highest sensitivity and high binding specificity.

Thus far, there are no good examples on use of MIPs in connection to monitoring of specific DNA sequences. Since the potential is large, one can expect such developments to happen in the near future.

### 3.3. Cell Detection

Jantra et al. [17] developed a label-free affinity biosensor for detecting and enumerating total bacteria based on the interaction between *E. coli* and Concanavalin A (Con A) immobilized on a modified gold surface. The analyses were completed in less than 20 min with both SPR and impedimetric capacitive biosensor. Compared to SPR (LOD: 6.1 × 10^7^ CFU·mL^−1^), the capacitive system showed much higher sensitivity (LOD: 12 CFU·mL^−1^). The developed system might be used successfully for total bacteria analysis from water sources.

Rydosz et al. [27] developed a new type of label-free microwave sensor in a form of interdigitated capacitor for bacterial lipopolysaccharide detection. The sensor surface was coated with T4 phage gp37 adhesin. The adhesin molecule bound *E. coli* by recognizing its bacterial host lipopolysaccharide (LPS). The binding was highly specific and irreversible. Recognition between the phage adhesion and bacterial LPS was based on the recognition of saccharide determinants of LPS which means very specific determination of bacterial strain or its endotoxins within the genus and the species. The selectivity experiments showed that the response for specific LPSs was significantly different from the reference measurements and the response for the non-specific LPSs was very close to the reference values. The developed method was promising for label-free LPS detection and could be used as an alternative for fiber-optic, electrochemical and classic biochemical and immunochemical methods. In their other study [28], same authors used bacteriophage-adhesin-coated long-period gratings for recognition of bacterial lipopolysaccharides. Long-period gratings (LPG) bio-functionalization methodology was based on coating the LPG surface with nickel ions which were capable of binding of gp37-histidine tag. The advantage of using adhesins for the bio-functionalization of the biosensor was to give ability for low-cost bio-sensitive molecule exchange and surface regeneration. In this work, for the first time, adhesion has been applied for bacteria and their endotoxin detection. T4 phage adhesin bound *E. coli* B LPS in its native or denatured form in a highly specific and irreversible way.

Rocha et al. [29] used alternating current electrokinetics (ACEK) capacitive sensing to detect and quantify the microbial cell abundance in aquatic systems. Microbial abundance was detected by measuring the electrical signal. Three different microbial cell cultures including *Bacillus subtilis*, *Alcanivorax borkumensis* and *Microcystis aeruginosa* were detected by using the developed system. The results showed that the sensor is capable of reliably detecting microbial cells even though they have major physiological differences between Gram-positive (*B. subtilis*), Gram-negative (*A. borkumensis*) and cyanobacteria (*M. aeruginosa*). The system is promising to detect and estimate microorganism population sizes in batch cultures, environmentally sourced seawater and groundwater systems.

For expanded use and commercialization of nanotechnology products, toxicity determination is an important field application. For this purpose, Qureshi et al. [18] developed a whole-cell based capacitive biosensor to determine the biological toxicity of nanoparticles (NPs). They used iron oxide (Fe_3_O_4_) nanoparticles as models in the study. The living *E. coli* cells were immobilized on the capacitive sensor chips. Then, these chips were interacted with different sizes of Fe_3_O_4_ NPs (5, 20 and 100 nm). The smallest Fe_3_O_4_ NPs resulted in a maximum capacitance change because they were able to interact with *E. coli* cells on the sensor chip very efficiently. The morphological changes on the surface of *E. coli* cells after interacting with Fe_3_O_4_ NPs were examined with SEM.

### 3.4. Heavy Metal Detection

Two metal binding proteins were over-expressed in *E. coli*, purified and immobilized on a thiol-modified capacitive sensor surface. Capacitive sensor was used to monitor conformational changes following heavy metal binding including copper, cadmium, mercury and zinc. Metal ion detection could be done in down to femtomolar concentrations with the developed system [30].

The same group expressed and purified metal resistance and metal regulatory proteins from bacterial strains and immobilized these proteins on the capacitive biosensor surface for heavy metal detection [19]. The system allowed the detection of heavy metals including Hg(II), Cu(II), Zn(II) and Cd(II) in pure solutions down to 10^−15^ M concentrations.

Corbisier at al. [20] established two different biosensor technologies for detection of several heavy metal ions in environmental samples. The principle of the first approach was to develop whole cell bacterial biosensors which emitted a bioluminescent/fluorescent signal in the presence of heavy metal ions. In the second approach, direct interaction between metal binding proteins and heavy metal ions was used as the detection principle in the capacitive biosensors. In the study, the main advantage of the whole cell based sensors was their ability to react only to biologically available metal ions, whereas the latter one (protein based sensors) was more sensitive towards metal ions.

MIPs selective for heavy metal ions have been presented in connection to separation technology [31,32,33,34,35,36]. It is an obvious development that also sensors for heavy metal ions will be developed based on selective MIPs.

### 3.5. Saccharide Detection

By using capacitive biosensors, Labib et al. [21] used the capacitive biosensor to detect glucose based on gold nanoparticles which were fixed on a poly-tyramine modified gold electrode surface. Dextran (MW: 39 kDa) was used as a regeneration agent by utilizing a competitive assay for glucose in the study. The dynamic range for glucose detection was between 1.0 × 10^−6^ and 1.0 × 10^−2^ M with a LOD value of 1.0 × 10^−6^ M.

**Figure 2:**
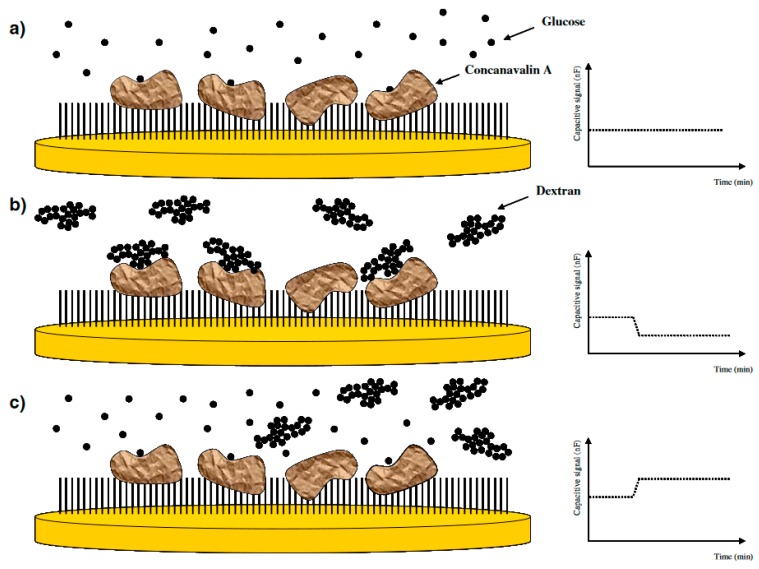
Schematic representation of the competitive glucose binding assay. (**a**) When glucose is injected into the capacitive system, it binds to the immobilized Concanavalin A (ConA) on the surface. However, this binding does not make any change in the capacitance level, as shown in the graph on the right, due to the small size of the glucose molecule; (**b**) When a glucose polymer (dextran) is injected into the system, binding of this big polymer to ConA results in a decrease in the capacitance signal; (**c**) When glucose is injected into the system again, displacement of dextran with glucose results in the capacitance turn back to the original baseline level. (Reproduced from Reference [22] with permission).

By using capacitive biosensors, the same authors [22] developed a technique based on the competition between a small molecular mass analyte and a large analyte-carrier conjugate. In the basis of the technique, when a large glucose polymer binds to the biorecognition molecule (Con A) immobilized on the electrode surface, it results in a decrease in the capacitance. Then, in the next step, when the low molecular mass analyte (glucose) is introduced to the system, the effect is reverse, the small glucose molecule will replace the large glucose polymer which is bound on the immobilized Con A as shown in Figure 2. By measuring the shift-back in capacitance, the glucose concentration could be determined by the technique. The authors used this technique to measure IgG as a glycoconjugate and detect its aggregation using immobilized Con A. When the glycoconjugate (IgG) was injected, the decrease in capacitance was measured to determine its concentration. In the second step, when concentrated glucose was injected into the system, the increase in capacitance was employed to determine the glucose concentration. The results showed that this technique is promising for monitoring small molecules with high sensitivity and broad detection range.

The radio frequency (RF) detection method is one of the promising methods for glucose detection out of several detection methods available [37]. When an analyte is injected into the RF biosensor, changes will occur owing to the inductive and capacitive effects [38]. These changes will cause losses and considerable shifts in the resonance frequency of the device. The change in capacitance is proportional to the dielectric constant and the distance between the biomolecule layer and the dielectric layer. A reusable robust RF biosensor was developed by Kim et al. [38] to monitor real-time glucose level in human serum. The resonance behaviour of the system was analysed with human serum samples containing different glucose concentrations ranging from 148–268 mg·dL^−1^, 105–225 mg·dL^−1^ and at a deionized water glucose concentration in the range of 25–500 mg·dL^−1^. The response time for glucose was measured as 60 s with a LOD value of 8.01 mg·dL^−1^. A total of 21 different experiments for each concentration of serum and D-glucose solution were analysed for reusability and the relative standard deviation (RSD) was less than 1% for each concentrations of serum samples and aqueous D-glucose solutions.

In the area of MIPs used for bioseparation, much work has been done concerning carbohydrates [39,40,41]. It is obvious that one can make good MIPs with high efficiency in binding target molecules or fragments thereof. Based on the observations from affinity chromatography, one can foresee that such systems will also soon be presented for capacitive biosensors [42,43,44,45,46,47,48] (Table 2).

### 3.6. Small Organic Molecules

**Figure 3:**
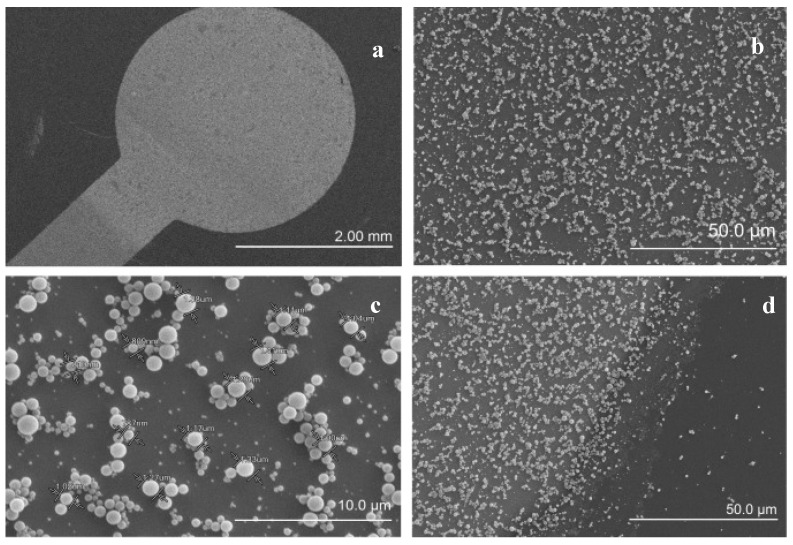
Scanning electron microscope (SEM) pictures of the electrode surface after functionalization with imprinted polymers. From left to right, top to bottom: (**a**) SEM picture of electrode surface; (**b**,**c**) SEM pictures of centre of the electrode; and (**d**) SEM picture of the border between the gold layer and wafer. (Reproduced from Reference [23] with permission).

Small molecules such as pesticides, herbicides, and antibiotics are widely discarded and encountered in naturally flowing waters. These pollutants in environment have an impact on communities and eco-systems. Therefore, detection of these molecules in a sensitive, cheap, robust and fast way is crucial. Lenain et al. [23] chose metergoline as a model compound representingsmall organic molecules such as pharmaceutical residues. Emulsion polymerization was used to produce small, uniformly sized, spherical MIPs. These MIP beads were attached to the poly-tyramine modified gold surface. Scanning electron microscope (SEM) images of the electrode surface are shown in Figure 3. Working range for metergoline was from 1.0 × 10^−6^ M to 50 × 10^−6^ M with a LOD value of 1.0 × 10^−6^ M. In cross reactivity analysis, even though the structural analogs showed binding, this contribution was only around 1.3 nF. The sensor response was more stable at higher ionic strength but the extent of capacitance change for different concentrations of analyte was less pronounced compared to lower electrolyte concentrations.

**Figure 4:**
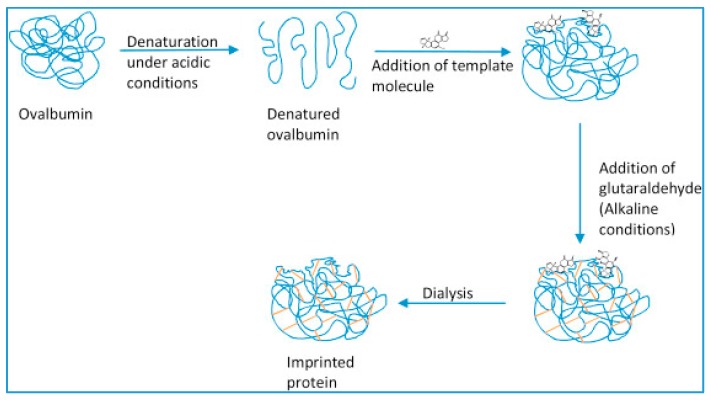
Schematic representation of bio-imprinting process. (Reproduced from Reference [24] with permission).

Bioimprinting is a technology used to mimic specific sites for modification of biological molecules. The process consists of four steps, as shown in Figure 4 [24].

(1) Unfolding the conformation of the starting protein under acidic conditions;
(2) Addition of template molecule and allow interaction between the template molecule and the denatured protein in order to form new molecular configurations;
(3) Cross-linking of the protein to stabilize the new molecular protein conformation; and
(4) Dialysis to remove the template molecule.

Gutierrez et al. [24] used bioimprinting to develop a capacitive biosensor for aflatoxin detection. Aflatoxins are natural food contaminants with a high risk for human health. Ovalbumin was used as platform for bioimprinting of aflatoxin because when bovine serum albumin (BSA) was used, there was no change in capacitance owing to the high hydrophobicity of sites of BSA. Three competitive mycotoxins were used in the cross-reactivity analysis and the changes in capacitance were significantly lower than that registered from aflatoxin solution.

Silicon nitride substrate (Si_3_N_4_) combined with magnetic nanoparticles (MNPs) was used to develop a capacitive immunosensor for ochratoxin A (OTA) detection. Silicon nitride allows an easy control of the film composition and thickness and also prevents the undesirable impurities. These are the main advantages of the substrate used in the study. Magnetic nanoparticles comprised of a conductive core and a carboxylic acid modified shell which was used to immobilize OTA antibodies. The LOD value was calculated as 4.57 × 10^−12^ M in the study and the selectivity results against ochratoxin B and aflatoxin G1 showed that the potential difference was not so significant when compared to the difference for OTA detection [25].

## 4. Molecular Imprinting

During the early 1970s, Wulff et al. [49] and Klotz et al. [50] introduced the molecular imprinting to imprint templates in organic polymers. Then, Mosbach et al. [51] reported the use of molecularly imprinted polymers (MIPs) in biosensors instead of antibodies which was a breakthrough.

The formation of MIPs involves three steps:

(1) Pre-complexation of functional monomers around the template molecule in solution either by forming covalent bonds or by self-assembling with non-covalent bonds;
(2) Polymerization of the resulting complex in the presence of cross-linking monomers and suitable solvents/ionic liquids as porogens; and
(3) Removal of template molecule from the synthesized polymer.

**Figure 5:**
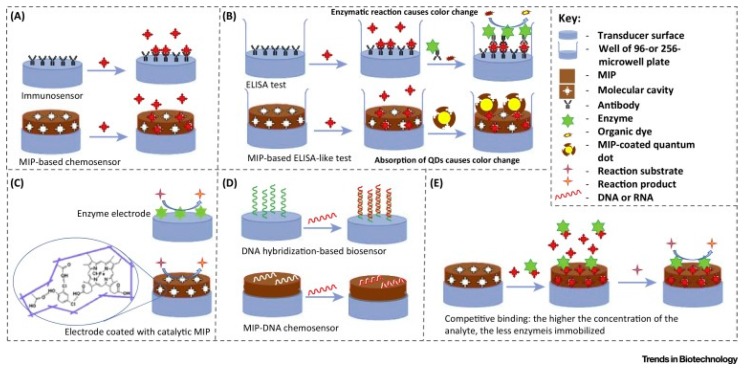
Different applications of MIPs in: (**A**) immunosensors; (**B**) enzyme-linked immunosorbent assay (ELISA); (**C**) enzyme electrodes, reaction rate and analyte concentration of enzyme electrodes and catalytic MIP-coated electrodes can be estimated by electroactive substrate/product consumption/production during the catalytic reaction or electron transfer from the electrode surface to the active centre of enzyme/MIP; (**D**) DNA chips; and (**E**) enzyme immobilization and competitive binding of the analyte. (Reproduced from Reference [52] with permission).

The resulting MIP contains recognition cavities capable of selective recognition of compounds that fit these cavities with respect to shape, size, position and orientation of the recognition sites [52]. How MIPs can mimic natural recognition units in different applications are shown schematically in Figure 5.

MIP technology has successfully been used for imprinting of low molecular weight templates. However there are still some difficulties of molecular imprinting technique when it is used for macromolecular templates including proteins. Due to this, many researchers have focused on the alternative techniques including imprinting the template directly onto a substrate or immobilizing the template protein on a glass support and use it as a protein stamp. The latter is called microcontact imprinting.

## 5. Microcontact Imprinting

Microcontact imprinting technique was first introduced by Chou et al. [53]. In the study, the authors formed the microcontact imprints between two cleaned glass surfaces. Template protein was immobilized on the cover slip and then, functional monomer was added on top in order to allow site-specific organization of the functional monomer by the template. In the next step, a drop of solution including cross-linker and initiator was dropped on the pre-modified support glass. Support glass was brought into contact with the cover slip to provide the two functionalized surfaces to contact. The glass assemblies were placed in a UV reactor to initiate the polymerization and it continued for 17 h. After polymerization, the cover slip was removed from the surface with forceps and the support was washed with various solutions. Polymer film thickness measurements showed it to be around 10 µm. The imprinted polymers showed better selectivity for their native templates than competing proteins. By this study, it was reported that microcontact imprinting technique might be used successfully for relatively large proteins in biosensor applications for detection and quantification [53].

There are lots of advantages of the technique over conventional molecular imprinting technique, as described in previous reports [54,55]. Only a few microliters of monomer solution are enough to polymerize dozens of samples at the same time in the same polymerization batch. Therefore, the method is useful to imprint templates which are very expensive or available in limited amounts. The technique also avoids potential solubility and conformational stability problems encountered with macromolecular targets, especially proteins. This is because immobilized templates are used in the process rather than adding the template to the monomer solution which otherwise is the standard procedure. This is also an important advantage for the ease of the template removal step after polymerization [54,55].

## 6. Applications of Microcontact Imprinting Method with Capacitive Biosensors

Ertürk et al. used microcontact imprinting method to prepare capacitive biosensors for various applications, please see Table 3. In their first application, the authors used BSA as the model protein to prepare microcontact BSA imprinted capacitive biosensor [56]. In the first step, the authors prepared glass cover slips with immobilized protein. This entity was called protein stamp. The cover slips were first modified with 3-aminopropyl-triethoxysilane (APTES) to introduce amino groups on the surface and then with glutaraldehyde to modify these amino groups. Then, the cover slips were immersed in BSA solution overnight at 4 °C. The electrode surface was on the other hand modified with polytyramine and then acryloyl chloride to introduce reactive groups on the surface which would be involved in the subsequent polymerization process. For the microcontact imprinting of BSA onto the gold electrode, the monomer solution containing methacrylic acid (MAA) as the functional monomer and poly-ethyleneglycoldimethacrylate (PEGDMA) as the cross-linker was prepared and the initiator was added into this solution. The modified gold electrode was treated with this monomer solution and then the protein stamp was brought into contact with this monomer treated electrode. Polymerization was initiated under UV light and ended in 15 min. After polymerization, the cover slip was removed from the surface with forceps and the microcontact BSA imprinted electrode was rinsed with water. The measurements were performed with a capacitive sensor involving the microcontact imprinted electrode inserted in an automated flow injection system developed by Erlandsson et al. [57].

**Figure 6:**
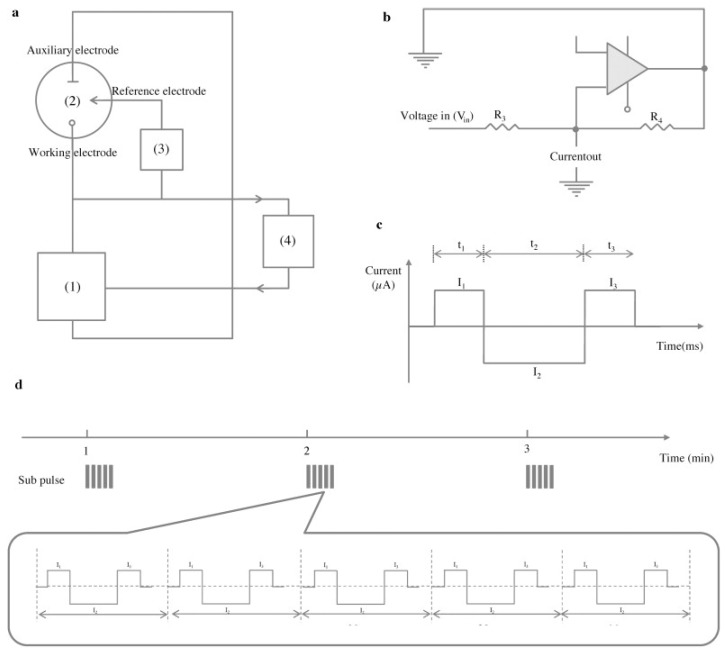
(**a**) Schematic representation of the capacitive system with current pulse method. The system is comprised of: (1) current source; (2) flow cell which is connected to the working, reference and auxiliary electrodes; (3) potential differential amplifier; and (4) a processor and ADC where the analogue potential is converted to digital signal; (**b**) A schematic view of Howland current pump used for supplying constant current; (**c**) Constant current supply to the sensor during the determined time periods to measure the resistance and capacitance; (**d**) Capacitance is measured every minute and each minute (pulse) contains five sub pulse measurements with 20 ms intervals. (Reproduced from reference [57] with permission).

Capacitive biosensors based on a potential pulse have been used in many applications [1,11,61,62] including microcontact imprinting as seen in Table 3. In this type of capacitive biosensors, a small potential pulse is applied to the working electrode and the capacitance is measured. This potentiometric pulse concept is sensitive to external electronic disturbances. Therefore, the system is prone to inaccurate measurements and poor baseline stability. The sharp potential pulse may induce damage to the surface of the working electrode after a while which results in decreasing response of the electrode and eventually to replacement of the working electrode with a new electrode [57]. Another alternative way is to use a current pulse method to measure the capacitance at the electrode/solution interface. Erlandsson et al. [57] developed a new concept to measure capacitance based on a constant current pulse to the biosensor transducer. In the basis of the principle, the system could be described as a simple resistor-capacitor (RC) circuit model. The schematic representation of the capacitance measurement via current pulse method is shown in Figure 6. The system consists of:

(1) A current source;
(2) An electro-chemical flow-cell which includes three electrodes: the working electrode which is a thin gold film coated with an insulating layer which functions as a bio-recognition layer to immobilize the ligand, the auxiliary and reference electrodes which are made from a platinum wire;
(3) A potential differential amplifier; and
(4) A processor which converts the analogue potential to digital signal.

**Figure 7:**
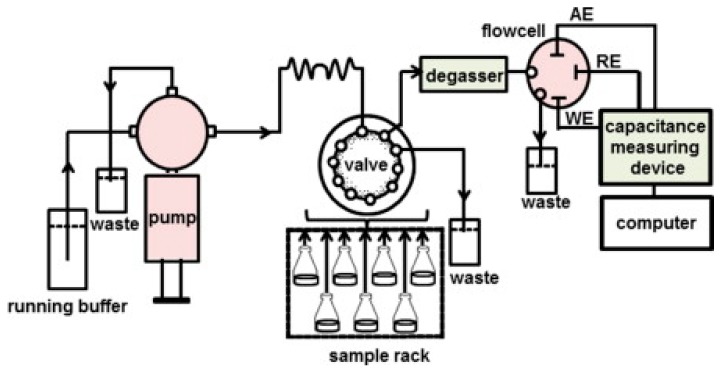
Schematic representation of automated flow injection capacitive system. The components shown in the figure are integrated into a box to make a single, portable unit. (Reproduced from Reference [63] with permission).

When using current pulse method, the capacitive measurements were performed with an automated flow-injection system as shown in Figure 7 [63].

In their next study, Ertürk et al. [58] used microcontact imprinting method for detection of an important biomarker, prostate specific antigen (PSA), for early detection of prostate cancer with capacitive biosensors. The standard solutions of different concentrations of PSA (2.0 × 10^−17^ M–2.0 × 10^−10^ M) were analysed by the system and the LOD value was calculated as 16 × 10^−15^ M. HSA and IgG were used as the competing proteins in order to test the selectivity of the system for PSA. These proteins are normally found in the levels of mg·mL^−1^ where PSA is found at approximately 4.0 ng·mL^−1^ in human serum. Therefore, when the developed system is tested against proteins at concentrations of 1 mg/mL which are normally found in one-million-fold higher concentrations, the system showed around two times more selectivity for PSA compared to HSA and IgG. In the next step, the sensitivity and the selectivity of the MIP system were compared with the performance of the system based on immobilized Anti-PSA antibody. The LOD was determined to be 12 × 10^−14^ M with the Anti-PSA system. The results showed that the MIP capacitive system was very promising to detect biomarkers which are important for diagnosis of various diseases. The authors compared the sensitivity of capacitive system for PSA detection with the microcontact imprinted surface plasmon resonance (SPR) system [64]. The LOD value was calculated around 91 pg·mL^−1^ (18 × 10^−14^ M) with the SPR biosensors. This result proves that the capacitive system is approximately 1000 times more sensitive than the SPR system.

Microcontact imprinting method was used for *E. coli* detection by Idil et al. [59]. The authors used a histidine containing specific monomer (N-methacryloyl-amido-histidine, MAH) as a metal chelating ligand. By using a meal-chelate between MAH and copper(II) (MAH-Cu^2+^), an enhanced selectivity against certain amino acid residues present on the cell wall of *E. coli* was achieved. N-(hydroxyethyl)methacrylate (HEMA) was used as a functional monomer to make a complex with MAH-Cu^2+^ (pHEMA-MAH-Cu^2+^) and EGDMA was used as cross-linker. The dynamic range for *E. coli* detection was between 1.0 × 10^2^ and 1.0 × 10^7^ CFU·mL^−1^ with a LOD value of 70 CFU·mL^−1^. *Bacillus subtilis*, *Staphylococcus aureus* and *Salmonella paratyphi* strains were used in selectivity experiments as competing strains. The cross-reactivity ratios were between 24% and 58% against pre-mixed suspensions of all bacterial strains. Even though the system showed cross-reactivity against competing strains, the ratio was negligible compared to the response of the system against *E. coli*. River water and apple juice were used to show the detection performance of the system from complex, real samples. Recovery value was found between 81% and 97% for *E. coli* detection from *E. coli* spiked (1.0 × 10^2^ to 1.0 × 10^4^ CFU·mL^−1^) river water samples. The sensor had potential to monitor *E. coli* in contaminated water or food supplies.

**Figure 8:**
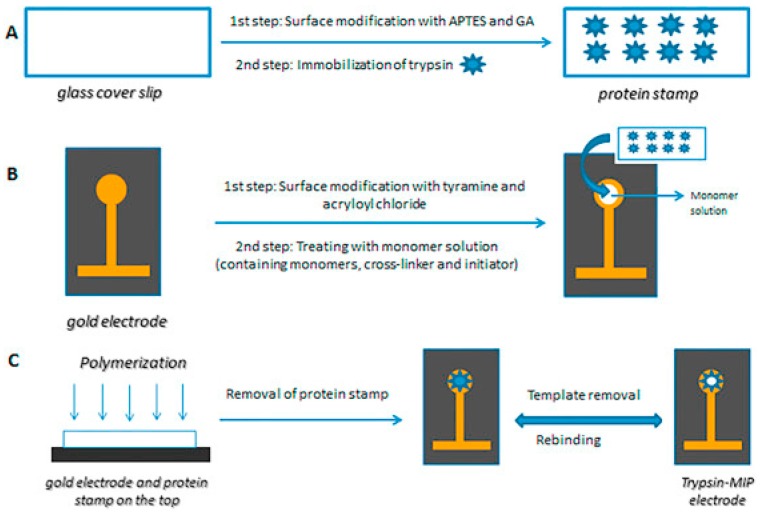
Schematic representation of preparation of trypsin imprinted capacitive electrodes using microcontact imprinting procedure: (**A**) preparation of glass cover slips (protein stamps); (**B**) preparation of capacitive gold electrodes; and (**C**) imprinting of trypsin to the electrode surface via microcontact imprinting method. (Reproduced from Reference [60] with permission).

Ertürk et al. [60] used microcontact imprinting method to develop capacitive biosensor for trypsin detection. A monomer solution containing hydroxymethylacrylamide, N-isopropylacrylamide (NIPAm), acrylamide and N,N-methylenebisacrylamide was prepared and N,N,N′,N′-tetramethylethylenediamine (TEMED, 5%, *v/v*) and ammonium persulfate (APS, 10%, *v/v*) were added into this solution. When the modified gold electrode was treated with monomer solution and brought into contact with the protein stamp which was carrying immobilized trypsin on top, the polymerization continued for 3–5 h at room temperature. Schematic representation of microcontact imprinting of trypsin on capacitive electrodes is shown in Figure 8. The dynamic range for trypsin detection was between 1.0 × 10^−13^ M and 1.0 × 10^−7^ M with a LOD value of 3.0 × 10^−13^ M. In order to test the selectivity and cross-reactivity of trypsin imprinted capacitive system for trypsin (MW 23.3 kDa, isoelectric point (pI): 10.1–10.5), chymotrypsin (chy) (MW: 25.6 kDa, pI 8.3), bovine serum albumin (BSA) (MW: 66.5 kDa, pI 4.7), lysozyme (lyz) (MW 14.3 kDa, pI 11.35) and cytochrome c (cyt c) (MW: 12.3 kDa, pI 10.0–10.5) were selected as competing agents. The concentrations of the proteins in the selectivity and cross-reactivity experiments were 1.0 mg·mL^−1^. If it was tested with the lower concentrations including 1.0 µg·mL^−1^ or 1.0 pg·mL^−1^, the selectivity results would be better because it would not be possible to detect interfering proteins in these concentration levels. Very low affinity of the system towards chy shows that the system can be used successfully for trypsin detection from pancreatic secretions where chy and try are found together. When the re-usability of the system was tested by monitoring the change in capacitance (-pF·cm^−2^) at the same concentration of trypsin (10^−7^ M), the loss in the detection performance was around 2% after 80 analyses and there was not any significant difference in the performance after storage for two months at 4 °C. In the last step, trypsin activity measured by capacitive system was compared with the trypsin activity measured by spectrophotometer at 410 nm. One unit of enzyme was defined as the amount of enzyme catalyzing the conversion of one micromole of substrate, N_α_-benzoyl-DL-arginine 4-nitroanilide hydrochloride (BAPNA), at 25 °C, pH: 8.1 per minute. The trypsin activity measured spectrophotometrically was 9 mU·mL^−1^ where the value was around 8.0 mU·mL^−1^ measured by capacitive system. The results showed that there was correlation between two methods. The advantages of the developed method including detection of trypsin in 20 min, high selectivity towards interfering proteins, high correlation with the spectrophotometer show that the system might be used successfully for detection of proteases and as a point of care system for diagnosis of pancreatic diseases.

## 7. Concluding Remarks

From the above, it is obvious that the combination of capacitive biosensors and MIPs offers great possibilities, both with regard to sensitive and selective assays as well as to stable measurements of extended periods of time. Sensitivity is now at a level where there are no needs to struggle for even more sensitive assays. What could be improved might be selectivity. It should however be mentioned that, with microcontact MIPs, higher selectivity was obtained as compared to that of monoclonal antibodies.

Cells and other particulate targets are still a bit difficult to assay. The number of CFU/mL that can be detected is far better than what can be reached with many of the methods used today. However, selectivity is too low and needs to be improved.

## Figures and Tables

**Table 1:** Different applications of capacitive biosensors developed for different targets.

	Target	Sensor Preparation Method	Dynamic range (M)	Limit of Detection (M)	Selectivity	Stability	Ref.
Proteins	Cholera toxin (CT)	Immobilization of anti-CT antibodies on self-assembled monolayer (SAM) of lipoic acid and 1-Ethyl-3-(3-dimethylaminopropyl)carbodiimide (EDC)	1.0 × 10^−13^ –1.0 × 10^−10^	1.0 × 10^−14^uiu	N/D	N/D	[9]
	Cholera toxin (CT)	Immobilization of anti-CT on gold nanoparticles incorporated on a poly-tyramine layer	0.1 × 10^−18^–10 × 10^−12^	9.0 × 10^−20^	N/D	Up to 36 times with an RSD of 2.5%	[10]
	HIV-1 p24 antigen	Immobilization of anti-HIV 1 p24 antigen on gold nanoparticles incorporated on a poly-tyramine layer	10.1 × 10^−20^–10.1 × 10^−17^	3.32 × 10^−20^	N/D	N/D	[11]
	VEGF	Immobilization of anti-VEGF aptamer first capturing the VEGF protein then, sandwiching with antibody-conjugated magnetic beads	13 × 10^−14^–2.6 × 10^−11^	N/D	N/D	N/D	[12]
Nucleic acids	25-mer oligo C	Covalent attachment of 25-mer oligo C on poly-tyramine modified electrode	10^−8^–10^−11^	10^−11^	Oligo-T was used as the competing agent, when the temperature was increased from RT to 50 °C, the ΔC value decreased from 48 nF·cm^−2^ to 3 nF·cm^−2^	N/D	[13]
	ssDNA	Thiol modified oligonucleotides were immobilized on Au and 3-glycidoxypropyl-tri-methoxy silane (GOPTS)	0.5 × 10^−6^–1.0 × 10^−3^	N/D	N/D	GOPTS functionalized surfaces were more stable at 4 °C. Ten-fold decrease in fluorescence intensity after 1 week even when the substrates were stored at 4 °C.	[14]
	Nampt	Immobilization of ssDNA aptamers on SAM of mercaptopropionic acid (MPA)	0–45 × 10^−10^	1.8 × 10^−11^	N/D	N/D	[15]
	Target DNA	Immobilization of pyrrolidinyl peptide nucleic acid probes (acpcPNA)	1.0 × 10^−11^–1.0 × 10^−10^	6–10 × 10^−12^	Complementary DNA provided a much higher ΔC compared to single and double mismatched DNA	Could be reused for 58–73 times with an average residual activity of ≥98%	[16]
Cells	Total bacteria	Based on the interaction between *E. coli* and concanavalin A immobilized on a modified gold surface	12 CFU·mL^−1^–1.2 × 10^−6^ CFU·mL^−1^	12 CFU·mL^−1^	N/D	For the first 35 cycles, the residual activity was 95% ± 3% (RSD = 3.2%). After 35 cycles, it was 85%.	[17]
	*E. coli*	*E. coli* cells immobilized on SAM of Mercaptopropionic acid (MPA)	8 × 10^5^ CFU·mL^−1^–8 × 10^7^ CFU·mL^−1^	N/D	N/D	N/D	[18]
Heavy metals	Hg(II), Cu(II), Zn(II), Cd(II)	Immobilization of metal resistance and metal regulatory proteins on gold electrode	10^−15^–10^−3^	N/D	N/D	N/D	[19]
	Cu(II), Cd(II), Hg(II)	1. Immobilization of whole bacterial cell to emit a bioluminescent/fluorescent signal in the presence of heavy metal ions	0–200 × 10^−6^	1.0 × 10^−6^	N/D	84% of the activity loss within 6 days	[20]
		2. Immobilization of heavy metal binding proteins	10^−15^–10^−1^			Stable over 16 days	
Saccharides	Glucose	Immobilization of ConA on gold nanoparticles incorporated on the tyramine modified gold electrode	1.0 × 10^−6^–1.0 × 10^−2^	1.0 × 10^−6^	Small sugars including D-fructose, D-mannose, D-maltose, methyl-α-D-glucopyranoside, methyl-α-D-mannopyranoside also bound instead of glucose	A neglectable loss in sensitivity after 10 cycles (7.5%)	[21]
	Glucose	Immobilization of ConA and replacement of small glucose with the large glucose polymer	1.0 × 10^−5^–1.0 × 10^−1^	1.0 × 10^−6^	Small molecules and high molecular weight dextran also bound instead of glucose	N/D	[22]
Small molecules	Metergoline	Immobilization of molecularly imprinted spherical beads on modified gold electrode	1–50 × 10^−6^	1.0 × 10^−6^	Cross reactant contribution was maximum 1.3 nF	N/D	[23]
	Aflatoxin B1	Bioimprinting	3.2 × 10^−6^–3.2 × 10^−9^	6.0 × 10^−12^	Competing agents’ binding was significantly lower than aflatoxin B1	Little variation over 28 injections with non-reduced Schiff’s bases	[24]
	Ochratoxin A (OTA)	Monoclonal anti-OTA immobilization on Si_3_N_4_ substrate combined with magnetic nanoparticles (MNPs)	2.47–49.52 × 10^−12^	4.57 × 10^−12^	Differences for ochratoxin B and aflatoxin B1 were not significant	N/D	[25]

**Table 2:** Molecularly imprinted polymers (MIPs) produced with high binding efficiency for affinity chromatography applications.

Template	Method	Matrix	Comments	Ref.
Benzo[a]pyrene (BAP)	BAP-imprinted poly (2-hydroxyethylmethacrylate-N-methacryloyl-(L)-phenylalanine composite cryogel cartridge	Aqueous solutions	Preconcentration on BAP with HPLC equipped with a fluorescence detector (HPLC-FLD)	[42]
Melamine	Melamine imprinted monolithic cartridges	Water + milk	MIP-solid phase extraction Extraction and enrichment of melamine	[43]
Cholesterol	Cholesterol imprinted polymeric nanospheres	Gastrointestinal mimicking solution	Cholesterol adsorption	[44]
Catalase	Iron chelated poly (2-hydroxyethylmethacrylate-N-methacryloyl-(L)-glutamic acid cryogel discs	Rat liver	Catalase purification from rat liver	[45]
L-phenylalanine (L-Phe)	L-Phe imprinted cryogel cartridges	Aqueous solutions	Chiral separation of l-phenylalanine with FPLC (fast protein liquid chromatography)	[46]
Triazine	Triazine imprinted monolithic columns	Aqueous solutions	Separation of triazine with capillary electro-chromatography (CEC)	[47]
Cytochrome c	Surface imprinted bacterial cellulose nanofibers	Rat liver	Cytochrome c purification from rat liver	[48]

**Table 3:** Capacitive biosensors developed for different targets using microcontact imprinting method.

Target	Biosensing Method	Monomers	Dynamic Range	LOD	Selectivity	Stability	Ref.
Bovine Serum Albumin (BSA)	Capacitive biosensor with current pulse method	Methacrylic acid (MAA); Poly ethyleneglycol-dimethacrylate (PEGDMA)	1.0 × 10^−20^ M–1.0 × 10^−8^ M	1.0 × 10^−19^ M	For human serum albumin (HSA): 5%; For IgG: 3%	>70 assays during 2 months	[56]
Prostate specific antigen (PSA)	Capacitive biosensor with current pulse method	MAA; EGDMA	2.0 × 10^−17^ M–2.0 × 10^−10^ M	16 × 10^−17^ M	Selectivity coefficient (k) = 2.27 for HSA, k = 2.02 for IgG	About same level during 50 injections	[58]
*E. coli*	Capacitive biosensor with current pulse method	HEMA; (2-Hydroxyethyl methacrylate), N-methacryloyl-L-histidine methyl ester (MAH), EGDMA	1.0 × 10^2^–1.0 × 10^7^ CFU·mL^−1^	70 CFU·mL^−1^	K = 3.14 for *B. subtilis*, k = 3.32 for *S. aureus*, k = 2.98 for *S. paratyphi*	About same level during 70 injections	[59]
Trypsin	Capacitive biosensor with current pulse method	N-isopropylacrylamide (NIPAm), N,N-methylenebisacryl, amide (MBAAm), Acrylamide, Hydroxymethylacrylamide	1.0 × 10^−13^ M–1.0 × 10^−7^ M	3.0 × 10^−13^ M	K = 733.1 for chymotrypsin (chy), k = 10.56 for BSA, k = 6.50 for lysozyme (Lyz), k = 3.46 for cytochrome c (cyt c)	The loss in performance was about 2% after 80 analyses	[60]
